# Practical and thermodynamic constraints on electromicrobially accelerated CO_2_ mineralization

**DOI:** 10.1016/j.isci.2022.104769

**Published:** 2022-07-16

**Authors:** Sabrina Marecos, Rae Brigham, Anastacia Dressel, Larissa Gaul, Linda Li, Krishnathreya Satish, Indira Tjokorda, Jian Zheng, Alexa M. Schmitz, Buz Barstow

**Affiliations:** 1 Department of Biological and Environmental Engineering, Cornell University, Ithaca, NY 14853, USA

**Keywords:** Microbiology, Biotechnology, Engineering, Energy sustainability

## Abstract

By the end of the century, tens of gigatonnes of CO_2_ will need to be removed from the atmosphere every year to maintain global temperatures. Natural weathering of ultramafic rocks and subsequent mineralization reactions can convert CO_2_ into ultra-stable carbonates. Although this will draw down all excess CO_2_, it will take thousands of years. CO_2_ mineralization could be accelerated by weathering ultramafic rocks with biodegradable lixiviants. We show that if these lixiviants come from cellulosic biomass, this demand could monopolize the world’s biomass supply. We demonstrate that electromicrobial production technologies (EMP) that combine renewable electricity and microbial metabolism could produce lixiviants for as little as $200 to $400 per tonne at solar electricity prices achievable within the decade. We demonstrate that EMP could make enough lixiviants to sequester a tonne of CO_2_ for less than $100. This work highlights the potential of this approach and the need for extensive R&D.

## Highlights

• Bio-production of acids to sequester 20 GtCO_2_ yr^−1^ could monopolize global agriculture
• Electromicrobial production could produce acids for as little as $200 to $400 per tonne
• Electromicrobial production could make acids to sequester 1 tonne CO_2_ for under $100

## Graphical abstract

**Figure:**
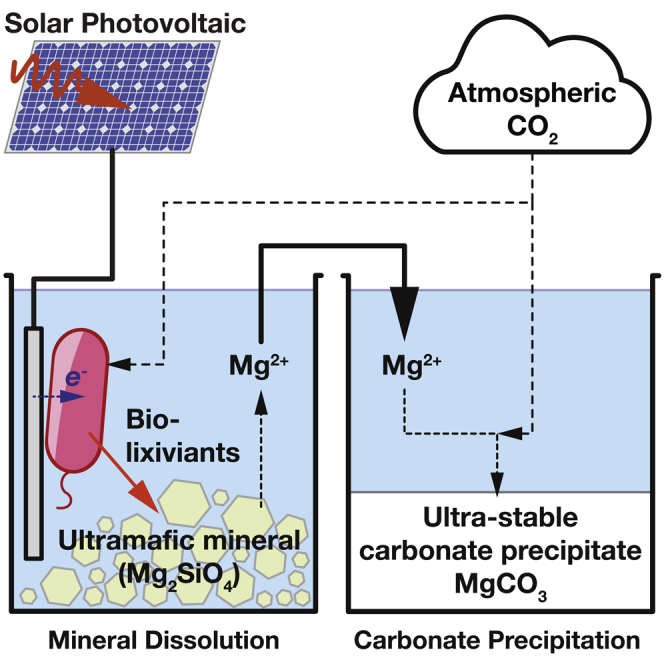

## Introduction

The IPCC’s (Intergovernmental Panel on Climate Change) 2018 special report on the impact of climate change highlighted the need for the significant deployment of negative emissions technologies (NETs) to limit global warming (Allen et al., 2019). The IPCC estimates that by the end of the 21st century, ≈ 20 gigatonnes of CO_2_ (GtCO_2_) will need to be removed from the atmosphere every year to limit global temperature rise to 1.5°C (Allen et al., 2019). In total, it is estimated that between ≈1,000 (Global Monitoring Laboratory, 2022) and 1,500 GtCO_2_ (Keller et al., 2018; Lackner and Azarabadi, 2021) will need to be removed from the atmosphere to restore it to its pre-industrial state. The US Department of Energy’s Carbon Negative Shot (Carbon Negative Shot) sets a target for the removal of gigatonnes of CO_2_ from atmosphere at a cost of less than $100 per tonne of CO_2_, a price point thought to be economical by the US National Academy of Sciences (Committee on Developing a Research Agenda for Carbon Dioxide Removal and Reliable Sequestration, 2019). However, no NET today has the right combination of cost, speed, capacity, perception of safety, and friendliness to agriculture (Committee on Developing a Research Agenda for Carbon Dioxide Removal and Reliable Sequestration, 2019).

Of all the negative emissions technologies examined for large-scale CO_2_ removal, carbon mineralization has the largest potential storage capacity (Beerling et al., 2020; Committee on Developing a Research Agenda for Carbon Dioxide Removal and Reliable Sequestration, 2019; Kelemen et al., 2019; Lehmann and Possinger, 2020). The CO_2_ storage capacity of carbon mineralization in ultramafic systems is truly enormous. Mafic materials are silicate minerals or igneous rocks that are rich in magnesium and iron. Ultramafic materials are typically composed of greater than 90% mafic material. Common examples of mafic rock-forming minerals include olivine, pyroxene, and amphibole while common mafic rocks include basalt, gabbro, and peridotite. Briefly, the silicate mineral (e.g., olivine) can break down into metal soluble metal ions (i.e., Mg^2+^ or Fe^2+^) and silica even in aqueous solvents at circumneutral pH (Power et al., 2013a). The metal ions can then react with CO_2_ dissolved in water from the atmosphere to form extremely long-lived carbonate minerals (Power et al., 2013a). For example, peridotite reservoirs across the globe (largely containing olivine) have the potential to mineralize and sequester 10^5^-10^8^ GtCO_2_ (Kelemen et al., 2019), between 100 and 100,000 × the excess CO_2_ in the atmosphere (≈1,000 to 1,500 GtCO_2_) (Global Monitoring Laboratory, 2022; Lackner and Azarabadi, 2021). Natural weathering (where the breakdown of the mineral occurs in rainwater) of exposed sections of mantle rocks will eventually draw down all excess CO_2_ in the atmosphere, but will take thousands of years to do it (Archer et al., 2009). Mineral grinding can accelerate the rate of weathering, but adds a cost of $100 to $300 per tonne of CO_2_ sequestered (Committee on Developing a Research Agenda for Carbon Dioxide Removal and Reliable Sequestration, 2019), at or above the Carbon Negative Shot’s $100 per tonne target (Carbon Negative Shot).

Mineral-dissolving microbes could accelerate mineral weathering (Committee on Developing a Research Agenda for Carbon Dioxide Removal and Reliable Sequestration, 2019; Power et al., 2010; Power et al., 2013b; Power et al., 2011) and reduce the need for cost mineral grinding. However, almost all mineral-dissolving microbes need to be powered by the degradation of plant biomass (*i.e*., the product of photosynthesis). For example, the mineral-dissolving microbe *Gluconobacter oxydans* B58 oxidizes the sugar glucose to the environmentally benign lixiviant (a mineral-dissolving compound) gluconic acid (glucose can be derived from the degradation of cellulose, one of the primary components of biomass) (Reed et al., 2016; Schmitz et al., 2021).

However, the world’s growing (Prosekov and Ivanova, 2018) and increasingly wealthy population (Hawksworth and Chan, 2015) are creating a growing need for arable land (Tilman et al., 2011), tightening the world’s biomass supply (Slade et al., 2014). Could the use of plant biomass to power CO_2_ mineralization compete with the world’s food supply?

Electromicrobial production (EMP) could enable the production of lixiviants for CO_2_ mineralization without competing with the world’s biomass supply. EMP technologies use specialized microorganisms that can absorb electricity (preferably renewable) into their metabolism to power CO_2_ fixation and the subsequent enzymatic production of chemicals. In theory, EMP could produce any compound that can be synthesized biologically, but we believe its most promising application is in the production of extremely high-volume, but low-cost chemicals such as biofuels (Salimijazi et al., 2020) and proteins (Leger et al., 2021; Wise et al., 2022).

EMP technologies (Claassens et al., 2019; Lips et al., 2018; Prévoteau et al., 2020; Rabaey et al., 2011; Rabaey and Rozendal, 2010; Salimijazi et al., 2019) that combine biological and electronic components have been demonstrated at lab scale to have the energy to chemical conversion efficiencies exceeding all forms of terrestrial photosynthesis (Haas et al., 2018; Liu et al., 2016), while theoretical predictions indicate that their efficiency could exceed all forms of photosynthesis (Claassens et al., 2019; Leger et al., 2021; Salimijazi et al., 2020; Wise et al., 2021). Globally, photosynthesis has an average solar to biomass conversion of less than 1% (Barstow, 2015). In contrast, lab-scale experiments have demonstrated a solar to product conversion efficiency of ≈10% for EMP (Liu et al., 2016), while theoretical predictions indicate that this could rise to over 15% (Salimijazi et al., 2020). This order of magnitude increase in solar to product conversion efficiency could allow the production of lixiviants with greatly reduced competition for arable land or wilderness.

However, at the time of writing EMP technologies are nascent, and difficult to implement even at lab scale. Our theoretical analyses of EMP (Salimijazi et al., 2020; Wise et al., 2022) are allowing us to assess which opportunities are the most fruitful to pursue and build support for pursuing them.

**Figure 1:**
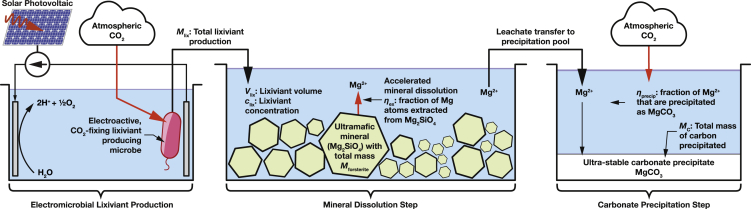
Overview of electromicrobially accelerated CO_2_ mineralization process Key parameters in this article are highlighted in this figure, Figure 2, and Tables 1 and 2.

In this article, we present a simplified model that estimates the global need for lixiviants for CO_2_ mineralization, the costs of synthesizing these lixiviants by electromicrobial production (see Figure 1 for an overview of this proposed system), and the costs of sequestering 1 tonne of CO_2_ using electromicrobially produced lixiviants.

## Results

A full set of symbols used in this article is included in Table 1.

**Table 1:** Symbols used in this article

Symbol	Unit	Description
*Ṅ*_lix_	molecule s^−1^	Lixiviant molecules produced per second by electromicrobial production system.
*N*_A_	Mol^−1^	Avogadro constant
F	An s Mol^−1^	Faraday constant
*P*_e, total_	J s^−1^	Total electrical power input into electromicrobial production system.
MW_lix_	g Mol^−1^	Molecular weight of the lixiviant molecule.
*E*	An s	Fundamental charge
*ν*_elix_	#	Number of electrons needed for the synthesis of a lixiviant molecule from CO_2_.
Δ*U*_cell_	V	Potential difference across bio-electrochemical cell.
*ν*_elix, add_	#	Number of electrons needed to convert a C_1_ compound to a lixiviant molecule.
*ν*_r_	#	Number of primary reduction products to make a molecule of the final product.
*ν*_er_	#	Number of electrons to reduce CO_2_ to a primary reduction product.
*ν*_Cr_	#	Number of carbon atoms per primary reduction product.
*ξ*_I2_	#	Faradaic efficiency of the bio-electrochemical cell.
*ξ*_I1_	#	Faradaic efficiency of the primary abiotic cell.
*ξ*_C_	#	Carbon transfer efficiency from cell 1 to cell 2.
*ν*_lix, NADH_	#	Number of NAD(P)H molecules needed to make a lixiviant molecule.
*ν*_p, Fd_	#	Number of Fd molecules needed to make a lixiviant molecule.
*ν*_p, ATP_	#	Number of ATP molecules needed to make a lixiviant molecule.
Δ*G*_ATP/ADP_	J	Free energy for regeneration of ATP
Δ*U*_membrane_	V	Inner membrane potential difference.
U_H2_	V	Standard potential of proton reduction to H_2_.
*U*_acceptor_	V	Standard potential of terminal electron acceptor reduction.
*U*_Q_	V	Redox potential of the inner membrane electron carrier.
*U*_NADH_	V	Standard potential of NADH
*U*_Fd_	V	Standard potential of Ferredoxin
*C*_Elix_	J g^−1^	Electrical energy cost per unit mass of lixiviant.
*C*_Slix_	¢ g^−1^	Solar energy cost per unit mass of lixiviant.
*V*_forsterite_	m^3^	Volume of forsterite needed to capture *M*_C_ of carbon.
*c*_lix_	Mol m^−3^	Concentration of lixiviant used to dissolve forsterite.
*V*_lix_	m^3^	Volume of lixiviant used to dissolve forsterite.
*ρ*_pulp_	#	Pulp density. Ratio of forsterite to lixiviant volumes.
*η*_precip_	#	Precipitation efficiency. Percentage of ions in leachate that are incorporated into magnesite.
*η*_ex_	#	Extraction efficiency. Percentage of Mg atoms in forsterite that are released into leachate solution.
*n*_C, olivine_	#	Maximum number of C atoms that can be sequestered per asymmetric unit of forsterite dissolved.
MW_forsterite_	g Mol^−1^	Molecular weight of forsterite (140.69).
Ζ	Mol m^−3^	Aggregated high uncertainty terms mass of lixiviant calculation.
*M*_lix_	g	Dry mass of lixiviant needed to sequester *M*_C_ of carbon as magnesite.
*M*_C_	g yr^−1^	Mass of C (not CO_2_) to be sequestered (10^13^ g yr^−1^). Multiply by 44/12 to calculate the mass of CO_2_.

**Table 2:** Electromicrobial lixiviant production model parameters

Parameter	Symbol	1. H_2_	2. EEU	3. H_2_ with Formate	4. EEU with Formate
**Electrochemical cell parameters**					

Input solar power (W)	*P*_*γ*_	1,000	1,000	1,000	1,000
Total available electrical power (W)	*P*_e, total_	330	330	330	330
CO_2_-fixation method		Enzymatic		Electrochemical	
Electrode to microbe mediator		H_2_	EEU	H_2_	EEU
Cell 1 cathode std. potential (V)	*U*_cell 1, cathode, 0_	N/A		0.82 (Torella et al., 2015)	
Cell 1 cathode bias voltage (V)	*U*_cell 1, cathode, bias_	N/A		0.47 (Liu et al., 2016)	
Cell 1 anode std. potential (V)	*U*_cell 1, anode, 0_	N/A		−0.43 (Yishai et al., 2017; Zhang et al., 2018)	
Cell 1 anode bias voltage (V)	*U*_cell 1, anode, bias_	N/A		1.3 (White et al., 2014)	
Cell 1 voltage (V)	Δ*U*_cell 1_	N/A		3.02	
Cell 1 Faradaic efficiency	*ξ*_I1_	N/A		0.8 (Rasul et al., 2019)	
Carbons per primary fixation product	*ν*_Cr_	N/A		1	
*e*^−^ per primary fixation product	*ν*_er_	N/A		2	
Cell 2 (Bio-cell) anode std. potential (V)	*U*_cell 2, anode, 0_	−0.41 (Torella et al., 2015)	−0.1 (Bird et al., 2011; Firer-Sherwood et al., 2008)	−0.41	−0.1
Bio-cell anode bias voltage (V)	*U*_cell 2, anode, bias_	0.3 (Liu et al., 2016)	0.2 (Ueki et al., 2018)	0.3	0.2
Bio-cell cathode std. potential (V)	*U*_cell 2, cathode, 0_	0.82			
Bio-cell cathode bias voltage (V)	*U*_cell 2, cathode, bias_	0.47			
Bio-cell voltage (V)	Δ*U*_cell 2_	2 (Liu et al., 2016)	1.59	2	1.59
Bio-cell Faradaic efficiency	*ξ*_I2_	1.0			

**Cellular electron transport parameters**					

Membrane potential difference (mV)	Δ*U*_membrane_	140 (SA in Figures S1 and S2 in Salimijazi et al., 2020)		140 (SA in Figures S1 and S2 in Salimijazi et al., 2020)	
Terminal *e*^−^ acceptor potential (V)	*U*_Acceptor_	0.82			
Quinone potential (V)	*U*_Q_	−0.0885 (Bird et al., 2011) (SA in Figure S5 in Salimijazi et al., 2020)		−0.0885 (Bird et al., 2011)	
Mtr EET complex potential (V)	*U*_Mtr_	N/A	−0.1 (SA in Figure S5 in Salimijazi et al., 2020)	N/A	−0.1 (Salimijazi et al., 2020)
No. protons pumped per *e*^-^	*p*_out_	Unlimited (SA in Figure S9 in Salimijazi et al., 2020)		Unlimited (Salimijazi et al., 2020)	

**Product synthesis parameters**					

No. ATPs for product synthesis	*ν*_p, ATP_	See Table S4			
No. NAD(P)H for product	*ν*_p, NADH_	See Table S4			
No. Fd_red_ for product	*ν*_p, Fd_	See Table S4			

Model parameters used in this article are based upon model parameters used in a previous analysis of the electromicrobial production of the biofuel butanol (Salimijazi et al., 2020). A sensitivity analysis (SA) that calculated the effect of varying key model parameters on the efficiency of product synthesis was performed in earlier work (Salimijazi et al., 2020). The location of these analyses (Salimijazi et al., 2020) is noted in the table above. EEU: Extracellular Electron Uptake.

### Simplified carbon mineralization reactions and lixiviant need

How much lixiviant is required to capture 20 GtCO_2_ per year (the approximate quantity estimated by the IPCC in order to limit global temperature rise to ≈1.5°C (Allen et al., 2019))? To simplify the calculation, we consider just the conversion of magnesium olivine (forsterite) into magnesium carbonate (magnesite) through a two-step reaction. In the first step, solid forsterite is dissolved into aqueous (aq) magnesium ions (Power et al., 2013a),

(Equation 1) $${\text{Mg}}_{2}{\text{SiO}}_{4,\text{s}}+4{\text{H}}_{\text{aq}}^{+}\to 2{\text{Mg}}_{\text{aq}}^{2+}+{\text{H}}_{4}{\text{SiO}}_{4}.$$

This dissolution reaction can occur at ambient temperature and in aqueous conditions (Oelkers et al., 2018). However, the rate of dissolution is the surface area limited and poses a significant speed limit in carbon mineralization (Oelkers et al., 2018).

In a later precipitation reaction, these Mg^2+^ ions react with atmospheric CO_2_ and precipitate as stable solid (s) carbonates including magnesite (MgCO_3_) (Power et al., 2013a),

(Equation 2) $${\text{Mg}}_{\text{aq}}^{2+}+{\text{CO}}_{3,\text{aq}}^{2-}\to {\text{MgCO}}_{3,\text{s}}.$$

This precipitation reaction can also occur under laboratory conditions (Power et al., 2013a), and is limited by the rate of equilibration of CO_2_ into water.

This article focuses purely on the acceleration of the dissolution reaction in (Equation 1) by lixiviants produced by EMP. In this article, we consider the upper limits of performance of an engineered microbe producing these lixiviants. At the time of writing, this microbe does not exist. The purpose of this work is to establish if it is even worth attempting to build such a microbe. That being said, naturally occurring acetogenic microbes (i.e., microbes that produce acetic acid (a biolixiviant)) can achieve conversion of electricity and CO_2_ to acetic acid with Faradaic efficiencies exceeding 90% (Prévoteau et al., 2020).

How much forsterite needs to be dissolved to capture 20 GtCO_2_? The maximum number of CO_2_ molecules (or C atoms) that can be sequestered by the dissolution of a single asymmetric unit of forsterite (Mg_2_SiO_4_), *n*_C, forsterite_, is 2 (one asymmetric unit of forsterite contains 2 Mg atoms, which can each react with one carbon atom). The molecular weight of a single forsterite asymmetric unit is 141 g per mole, and the molecular weight of 2C atoms is 24 g per mole. Thus, the minimum mass of forsterite needed to capture a mass of carbon *M*_C_ (e.g., 0.27 GtC corresponding to one GtCO_2_), is,

(Equation 3) $$\frac{M_{\text{forsterite}}}{M_{\text{C}}}=\frac{{\text{MW}}_{\text{forsterite}}}{{\text{MW}}_{\text{C }}n_{\text{C},\text{ forsterite}}}.$$

Therefore, to sequester 1 gigatonne of CO_2_, at least 16 gigatonnes of forsterite need to be dissolved (Power et al., 2013a).

How much lixiviant is needed to dissolve this much forsterite? The volume of the forsterite can be simply calculated from its density, $\rho_{\text{forsterite}}$,

(Equation 4) $$V_{\text{forsterite}}=M_{\text{forsterite}}/\rho_{\text{forsterite}}.$$

The volume of the lixiviant, *V*_lix_, can be calculated from the experimentally derived pulp density (the mass, in grams, of solid dissolved per 100 mL of solution) that gives the best mineral dissolution,

(Equation 5) $$\rho_{\text{pulp}}=M_{\text{forsterite}}/V_{\text{lix}}.$$

*ρ*_pulp_ is typically expressed in % w/v. For example, $\rho_{\text{pulp}}=2\%$, means that 2 g of forsterite are dissolved in 100 mL of lixiviant. However, so that we can use the experimentally derived pulp density along with our preferred units, we express *ρ*_pulp_ in terms of g m^−3^ (simply multiply *ρ*_pulp_ in % w/v by 10^4^).

The mass of the dry lixiviant can be calculated simply from its molecular weight; concentration, *c*_lix_; and volume, *V*_lix_,

(Equation 6) $$M_{\text{lix}}={\text{MW}}_{\text{lix}}\;c_{\text{lix}}\;V_{\text{lix}}.$$

A full listing of molecular weights of the lixiviant compounds considered in this article is included in Table S1.

Thus, the minimum mass of the lixiviant needed to dissolve *M*_forsterite_, and hence to sequester *M*_C_ is,

(Equation 7) $$M_{\text{lix}}\geq \frac{M_{\text{C }}{\text{MW}}_{\text{forsterite }}c_{\text{lix}}{\text{ MW}}_{\text{lix}}}{{\text{MW}}_{\text{C}}\;n_{\text{C},\text{ forsterite}\;}\rho_{\text{pulp}}}.$$

However, not all steps in the CO_2_ mineralization process will be perfectly efficient. The extraction of Mg from forsterite will be imperfect (Equation 1), as will the later precipitation of Mg^2+^ ions as a carbonate (Equation 2). To account for this, we introduce extraction efficiency, $\eta_{\text{ex}}$, and precipitation efficiency, $\eta_{\text{precip}}$,

(Equation 8) $$M_{\text{lix}}=\frac{M_{\text{C}}\;{\text{MW}}_{\text{forsterite }}c_{\text{lix}\;}{\text{MW}}_{\text{lix}}}{{\text{MW}}_{\text{C}}\;n_{\text{C},\text{forsterite}}\;\eta_{\text{ex}}\;\eta_{\text{precip}}\;\rho_{\text{pulp}}}.$$

The formula for the mass of lixiviant, *M*_lix_, required to sequester a given amount of carbon per year, is composed of two sets of terms: those with at least reasonably well-known values (MW_forsterite_, MW_C_, *n*_C, forsterite_), and a second set whose values have high uncertainty (*η*_ex_, *η*_precip_, *ρ*_pulp_, *c*_lix_),

(Equation 9) $$M_{\text{lix}}=\frac{M_{\text{C}}\;MW_{\text{forsterite}\;}{\text{MW}}_{\text{lix}}}{\underset{\text{high certainty terms}}{\underset{︸}{{\text{MW}}_{\text{C}}\;n_{\text{C},\text{ forsterite}}}}}\times \frac{c_{\text{lix}}}{\underset{\text{high uncertainty terms},\;\zeta }{\underset{︸}{\eta_{\text{ex}}\;\eta_{\text{precip}\;}\rho_{\text{pulp}}}}}.$$

We denote the product of the high uncertainty terms as ζ, the inverse CO_2_ mineralization performance. The higher ζ gets, the more lixiviant it takes to sequester *M*_C_. Given that the uncertainty in each of the four terms in ζ is equally high, we choose to make our estimate of *M*_lix_ a function of ζ rather than any single uncertain parameter. Thus,

(Equation 10) $$M_{\text{lix}}=\frac{M_{\text{C}}{\text{ MW}}_{\text{forsterite}}{\text{ MW}}_{\text{lix}}}{{\text{MW}}_{\text{C}}\;n_{\text{C},\text{ forsterite}}}\zeta .$$

Parameterizing the estimate of *M*_lix_ in this way does not reduce uncertainty, but does let us assess the consequences of different values of ζ, ranging from a very optimistic value (where mineralization performance is high) all the way up to a value of ζ that is so high that all of the biomass that the world makes in a year has to be turned into lixiviants (see Results for further discussion of this).

### Theory of electromicrobial production

We have extended our theoretical framework for calculating the efficiency of EMP (Salimijazi et al., 2020; Wise et al., 2021) to calculate the energy cost of lixiviant production from renewable electricity and CO_2_. Full derivations of the equations presented here can be found in the supplement to our original electromicrobial production efficiency theory article (Salimijazi et al., 2020), and in our recent work on the electromicrobial production of protein with extends our theory to calculate the energy (electrical or solar) costs of producing a gram of product (Wise et al., 2021).

**Figure 2:**
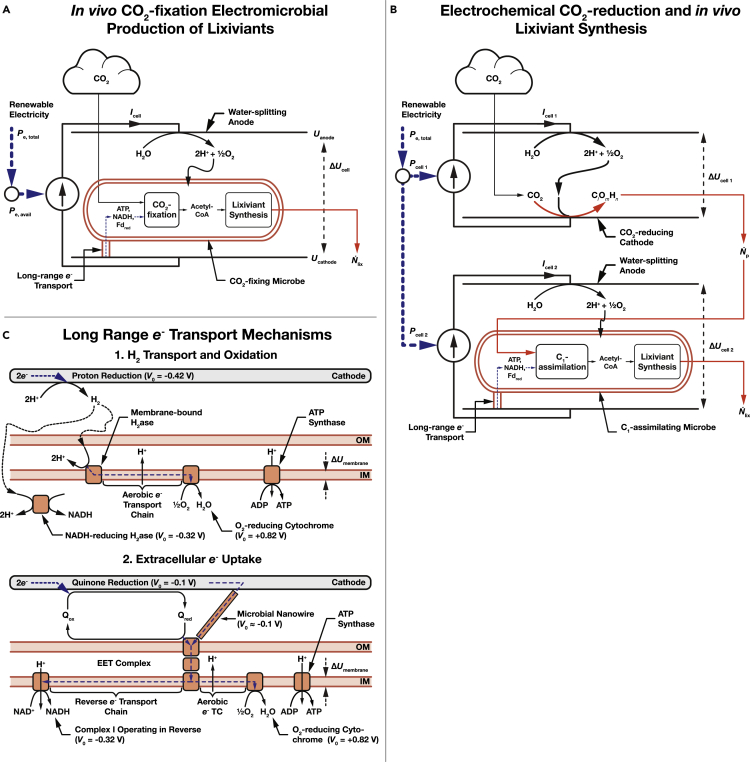
Schematic of the electromicrobial production of lixiviants for CO_2_ mineralization (A) Single bio-electrochemical cell system where electricity is used to power *in vivo* CO_2_ - and subsequent lixiviant synthesis. (B) Dual electrochemical cell system where CO_2_ is reduced in the first cell, and then assimilated in the second cell to produce lixiviant molecules. (C) Long-range *e*^−^ transfer mechanisms considered in this article. In the first, H_2_ is electrochemically reduced on a cathode, transferred to the microbe by diffusion or stirring, and enzymatically oxidized. In the second mechanism, extracellular electron uptake (EEU), *e*^−^ are transferred along a microbial nanowire (part of a conductive biofilm), or by a reduced medium potential redox shuttle such as a quinone or flavin, and are then oxidized at the cell surface by the extracellular electron transfer (EET) complex. From the thermodynamic perspective considered in this article, these mechanisms are equivalent. Electrons are then transported to the inner membrane where reverse electron transport is used to regenerate NAD(P)H, reduced Ferredoxin (not shown), and ATP (Rowe et al., 2018, 2021). Parameters for these systems are shown in Table 2.

We consider a bio-electrochemical system used to deliver electrons to microbial metabolism (Figure 2B). Electrical power is used to generate lixiviant molecules with a molecular weight MW_lix_. The amount of electricity needed to produce a unit-mass of the lixiviant is,

(Equation 11) $$C_{\text{Elix}}\geq N_{\text{A}}\;\Delta U_{\text{cell }}e\nu_{\text{elix}}/MW_{\text{lix}},$$

where *eν*_elix_ is the amount of charge needed to synthesize a single lixiviant molecule from CO_2_ (the fundamental charge, *e*, multiplied by the number of electrons needed for synthesis, *ν*_elix_); Δ*U*_cell_ is the potential difference across the bio-electrochemical cell; and *N*_A_ is the Avogadro constant. A derivation of (Equation 11) can be found in Wise et al., 2021, building upon derivations in Salimijazi et al., 2020.

For systems where CO_2_ reduction is performed electrochemically, and the resulting reduction product (typically a C_1_ compound such as formic acid) (Appel et al., 2013; White et al., 2014, 2015) is further reduced enzymatically, *ν*_elix_ is substituted for the number of electrons needed to convert the C_1_ product into the lixiviant, *ν*_elix, add_ (Salimijazi et al., 2020),

(Equation 12) $$C_{\text{Elix}}\geq \frac{e\nu_{\text{elix},\text{ add}}\;N_{\text{A}}\left(\Delta U_{\text{cell}1}\left(\frac{\nu_{\text{r }}\nu_{\text{er}}\;\nu_{\text{Cr}}\;\xi_{\text{I}2}}{\xi_{\text{I}1}\;\xi_{C\;}\nu_{\text{elix},\text{ add}}}\right)\;+\;\Delta U_{\text{cell}2}\right)}{MW_{\text{lix }}\xi_{\text{I}2}},$$

where *ν*_r_ is the number of primary reduction products (i.e., formic acid molecules) needed to synthesize a molecule of the final product, *ν*_er_ is the number of electrons needed to reduce CO_2_ to a primary reduction product (i.e., two in the case of formic acid), *ν*_Cr_ is the number of carbon atoms per primary fixation product (i.e., one in the case of formic acid), *ξ*_I2_ is the Faradaic efficiency of the bio-electrochemical cell, *ξ*_I1_ is the Faradaic efficiency of the primary abiotic cell 1, *ξ*_C_ is the carbon transfer efficiency from cell 1 to cell 2. A derivation of (Equation 12) can be found in Wise et al., 2021.

We calculate the electron requirements for lixiviant synthesis, *ν*_elix_ (from CO_2_) or *ν*_elix, add_ (from an electrochemical CO_2_ reduction product), from the number of NAD(P)H (*ν*_lix, NADH_) reduced Ferredoxin (Fd_red_; *ν*_lix, Fd_) and ATP (*ν*_lix, ATP_) molecules needed for the synthesis of the molecule, along with a model of the mechanism used for electron delivery to the microbe (Salimijazi et al., 2020).

For systems that rely on H_2_-oxidation for electron delivery such as the Bionic Leaf (Liu et al., 2016; Torella et al., 2015),

(Equation 13) $$\nu_{\text{elix},{\text{H}}_{2}}=2\nu_{\text{lix},\text{NADH}}+2\nu_{\text{lix},\text{Fd}}+\nu_{\text{lix},\text{ATP}}\frac{\text{ceil}\left(\text{Δ}G_{\text{ATP}/\text{ADP}}/e\text{Δ}U_{\text{membrane}}\right)}{\text{floor}\left(\left(U_{{\text{H}}_{2}}-U_{\text{acceptor}}\right)/\text{Δ}U_{\text{membrane}}\right)}$$

where Δ*G*_ATP/ADP_ is the free energy required for the regeneration of ATP, Δ*U*_membrane_ is the potential difference across the cell’s inner membrane owing to the proton gradient, *U*_H2_ is the standard potential of proton reduction to H_2_, *U*_acceptor_ is the standard potential of terminal electron acceptor reduction (typically O_2_ + 2*e*^−^ to H_2_O), the ceil function rounds up to the nearest integer, and the floor function rounds down to the nearest integer. A full derivation of Equation 13 can be found in Section 2 (Equations 10, 11, 12, 13, 14, 15, 16, 17, 18, 19, and 20) of the supplement in Salimijazi et al., 2020.

The transmembrane potential difference, Δ*U*_membrane_, is the largest source of uncertainty in this calculation. Therefore, we present a range of efficiency estimates in Figure 4 and throughout the text for Δ*U*_membrane_ = 80 mV (BioNumber ID (Milo et al., 2010) (BNID) 10,408,284) to 270 mV (BNID 107135), with a central value of 140 mV (BNIDs 109,774, 103,386, and 109,775). The upper error bars in Figure 4 correspond to Δ*U*_membrane_ = 240 mV, the lower bars correspond to Δ*U*_membrane_ = 80 mV, and the center of the bar corresponds to Δ*U*_membrane_ = 140 mV.

For systems that rely on EEU for electron delivery such as *Shewanella oneidensis* (Rowe et al., 2021; Salimijazi et al., 2020),

(Equation 14) $$\begin{array}{ll} \nu_{\text{elix},\text{EEU}}= & 2\nu_{\text{lix},\text{ NADH}}+2\nu_{\text{lix},\text{Fd}}\\ & +\;\nu_{\text{lix},\text{ATP}}\frac{\text{ceil}\left(\text{Δ}G_{\text{ATP}/\text{ADP}}/e\text{Δ}U_{\text{membrane}}\right)}{\text{floor}\left(\left(U_{\text{Q}}-U_{\text{acceptor}}\right)/\text{Δ}U_{\text{membrane}}\right)}\\ & +\;\nu_{\text{lix},\text{NADH}}\frac{\text{ceil}\left(\left(U_{\text{NADH}}-U_{\text{Q}}\right)/\Delta U_{\text{membrane}}\right)}{\text{floor}\left(\left(U_{\text{Q}}-U_{\text{acceptor}}\right)/\Delta U_{\text{membrane}}\right)}\\ & +\;\nu_{\text{lix},\text{Fd}}\frac{\text{ceil}\left(\left(U_{\text{Fd}}-U_{\text{Q}}\right)/\text{Δ}U_{\text{membrane}}\right)}{\text{floor}\left(\left(U_{\text{Q}}-U_{\text{acceptor}}\right)/\text{Δ}U_{\text{membrane}}\right)}, \end{array}$$

where *U*_Q_ is the redox potential of the inner membrane electron carrier, thought to be ubiquinone (Rowe et al., 2018), *U*_NADH_ is the standard potential of NAD(P)H, and *U*_Fd_ is the standard potential of Ferredoxin. A full derivation of Equation 14 can be found in Section 7 (Equations 77 to 91) of the supplement in Salimijazi et al., 2020.

The NAD(P)H, ATP, and Fd_red_ requirements for lixiviant synthesis were calculated by balancing networks of reactions for the autotrophic synthesis of the molecule from CO_2_ or formate (COOH^−^). We enumerated all reaction steps for the production of four environmentally benign lixiviant molecules (acetic, citric, 2,5-diketo-gluconic, and gluconic acid) from acetyl-CoA and using data from the KEGG database (Kanehisa, 2019; Kanehisa et al., 2021; Kanehisa and Goto, 2000) in Table S2.

Lixiviant synthesis reactions were complemented with reactions for CO_2_-fixation and C_1_-assimilation. For this article, we considered six scenarios in which CO_2_ was fixed by the well-known Calvin cycle (Berg et al., 2002), the Reductive Tricarboxylic Acid cycle (Alissandratos and Easton, 2015; Claassens et al., 2016), Wood-Ljungdahl (WL) Pathway (Berg et al., 2002); the 3-hydroxypropionate/4-hydroxybutyrate (3HP-4HB) Pathway (Berg et al., 2007; Claassens et al., 2016); 3-hydroxypropionate (3HP) Cycle (Zarzycki et al., 2009); and the Dicarboxylate/4-hydroxybutyrate (4HB) Cycle (Huber et al., 2008). In addition, we also considered the artificial Formolase formate assimilation pathway (Siegel et al., 2015). These reactions can be found in Table S3.

The CO_2_-fixation and C_1_-assimilation and lixiviants were combined by hand into a set of stoichiometric matrices), **S**_**lix**_, for each reaction network. Stoichiometric matrices are included in Data S1. Stoichiometric matrices were balanced with a custom flux balance program (Barstow, 2021) to find the overall stoichiometry for the synthesis of each lixiviant using each CO_2_-fixation or C_1_-assimilation pathway. The balanced overall stoichiometry for the synthesis of each lixiviant by each CO_2_ fixation or C_1_ assimilation pathway can be found in Table S4.

### Mass of lixiviants needed for global scale CO_2_ sequestration can outstrip global supply when de-mineralization efficiencies are low

**Figure 3:**
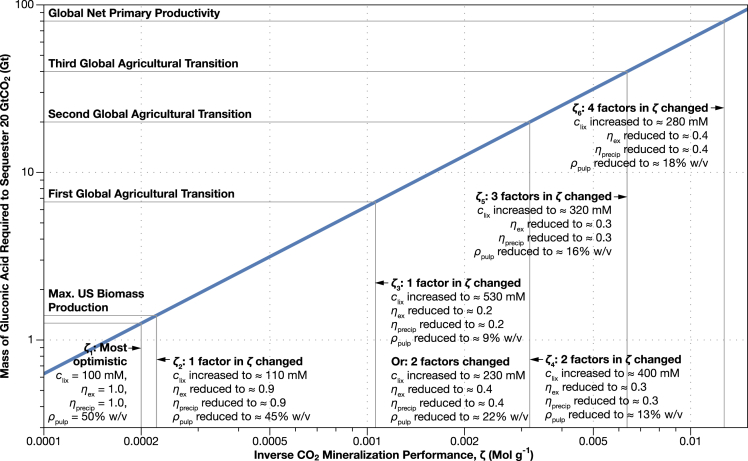
Accelerated mineralization could require hundreds of millions to tens of billions of tonnes of lixiviants per year If these lixiviants were produced from cellulosic biomass, this could put a significant strain on the world agricultural system. We calculated the mass of lixiviant (*M*_lix_) needed to accelerate the forsterite dissolution step of the mineralization of 20 GtCO_2_ per year using Equation 10 as a function of the inverse CO_2_ mineralization performance, *ζ*, the combination of the most uncertain parameters in our estimate of lixiviant mass. We chose to display results for gluconic acid as it has the highest molecular weight and provides an upper bound on the lixiviant mass requirement. Our most optimistic estimate for *ζ* (*ζ*_1_) is shown as the left most vertical line on the plot. The second marked value of *ζ* (*ζ*_2_) corresponds to a mass of lixiviant equal to all of the cellulosic biomass produced in the United States in a year. The third, fourth, and fifth lines (*ζ*_3_ to *ζ*_5_) correspond to increasing biomass withdrawals from the biosphere that come with increasingly severe consequences for agriculture and human society including the adoption of vegetarian diets, population control and widespread managed agriculture and forestry (Slade et al., 2014). The sixth (*ζ*_6_) and final line corresponds to the biomass production of the entire world in a year (net primary productivity). This plot can be reproduced with the nlixiviant.py code in the ElectroCO2 repository (Barstow, 2021).

We plot the mass of lixiviant required for the sequestration of 20 GtCO_2_ per year (the amount of CO_2_ that will need to be sequestered per year in the late 21^st^ century (Allen et al., 2019) as a function of the product of the inverse CO_2_ mineralization performance, ζ, in Figure 3.

What range of values could we expect for the CO_2_ mineralization efficiency? To estimate ζ we have made educated guesses for each of the values from the scientific literature. At the optimistic end of the spectrum, we assume that the concentration of lixiviant is 100 mM (corresponding to ≈ pH 2.1 for citric acid, pH 2.4 for gluconic acid, and pH 2.9 for acetic acid; STAR Methods), the extraction and the precipitation efficiency are both 100%, and the pulp density is 50% w/v (500,000 g m^−3^) (Macdonald, 2007),

(Equation 15) $$\begin{array}{l} \zeta_{\text{optimistic}}=100\;{\text{Mol m}}^{-3}/\left(1\times 1\times 5\times 10^{5}\;{\text{g m}}^{-3}\right)\\ =2\times 10^{-4}{\text{ Mol g}}^{-1}. \end{array}$$

The optimistic value of ζ is marked as the furthest left vertical line in Figure 3 and corresponds to a consumption of 1.26 Gt of dry lixiviant per year. Even this optimistic scenario corresponds to a significant amount of biomass, accounting for 90% of US biomass production (Perlack and Stokes, 2011) even if cellulosic biomass could be converted to lixiviant with 100% mass conversion efficiency.

What are the consequences for lixiviant demand if some of the factors included in *ζ* are slightly less than the optimistic estimates? If just the lixiviant concentration, *c*_lix_, increases by only 10%, or any one of the denominator factors in *ζ* (*η*_ex_, *η*_precip_, *ρ*_pulp_) decreases by 10%, the minimum mass of lixiviant required to sequester 20 GtCO_2_ will rise to 1.4 Gt, equal to the entire biomass production of the United States (Perlack and Stokes, 2011) (Figure 3, second vertical line from the left). The same increase in *ζ* can be achieved by a simultaneous 3% increase in *c*_lix_, and 3% reduction in *η*_ex_, *η*_precip_, and *ρ*_pulp_. We have calculated possible combinations of values of *c*_lix_, *η*_ex_, *η*_precip_, and *ρ*_pulp_ that produce each of the values of *ζ* highlighted in Figure 3 in Table S5.

What are the consequences for lixiviant demand if one or more of the factors in *ζ* are significantly less than the optimistic estimates? Slade et al., 2014) calculated the effects of withdrawing increasing quantities of bio-energy from the biosphere. We can make an approximate conversion from bio-energy to dry weight of biomass by dividing by the energy density of dry cellulosic material,

(Equation 16) $$M_{\text{biomass}}\approx \frac{E_{\text{biomass}}}{\rho_{\text{energy},\text{dry cellulose}}}.$$

Slade et al., 2014 identified three transition points with increasingly restrictive consequences for global civilization (including a combination of crop yield increases, and population, diet and forestry control) that come with increasing biomass use. We have marked these transition points as the third, fourth and fifth horizontal lines from the bottom of Figure 3. We have marked values of ζ that correspond to these transition points as the third, fourth, and fifth vertical lines from the left in Figure 3.

A significant change in one of the factors of *ζ* or two smaller simultaneous changes is required for lixiviant demand to pass the first consequential transition identified by Slade et al., 2014. The first transition occurs when the withdrawal of bio-energy from the biosphere exceeds 100 EJ per year (EJ) (corresponding to ≈7 Gt of dry biomass). Exceeding this withdrawal rate will require that crop yields keep pace with demand; and either adoption of vegetarian diets, or a low global population (<9 billion), or limited deforestation. Increasing the lixiviant demand rate to ≈7 Gt per year occurs when ζ rises to 1 × 10^−6^ Mol g-^1^. This increase in *ζ* will happen if *c*_lix_ rises by a factor of ≈5 to 530 mM, or a reduction in any one of the denominator factors (*η*_ex_, *η*_precip_, and *ρ*_pulp_) to ≈1/5^th^ of its optimistic value (Figure 3, Table S5). *ζ* can also rise to 10^−6^ Mol g^−1^ if *c*_lix_ rises by a factor of ≈2, and one of the denominator factors falls to ≈ ½ of its optimistic value or two of the denominator factors fall to ≈ ½ of their optimistic value. Alternatively, the same increase in *ζ* can also happen if *c*_lix_ increases by ≈ 50% (^3^/_2_), and the denominator factors all decrease to about ^2^/_3_^rds^ of their optimistic values (Table S5).

Significant changes in two factors contributing to *ζ* are required for lixiviant demand to pass the second consequential transition identified by Slade et al., 2014. This second transition occurs when the withdrawal of bio-energy from the biosphere exceeds 300 EJ per year (≈20 Gt of dry biomass per year). Exceeding this withdrawal rate will require that increases in crop yields outpace demand; and either adoption of vegetarian diets, a low population, or limited deforestation. Increasing the lixiviant demand rate to 20 Gt occurs if there are simultaneous reductions in two of the three denominator factors of *ζ* to ≈^1^/_4_^th^ of their optimistic value, or an increase in *c*_lix_ to ≈400 mM (a factor of 4) (Figure 3 and Table S5). Alternatively, a doubling of *c*_lix_ to ≈200 mM, and a reduction in all the denominator factors to ½ their optimistic value will also raise lixiviant demand to 20 Gt (Table S5).

Significant changes in three factors contributing to *ζ* are required for lixiviant demand to pass the third consequential transition identified by Slade et al., 2014. The third transition point occurs when bio-energy withdrawal exceeds 600 EJ yr^−1^ (≈40 Gt of dry biomass per year). Exceeding this withdrawal rate requires high input farming, high increases in crop yields, limiting the global population to <9 billion, and adoption of either vegetarian diets or managed forestry (Slade et al., 2014). Increasing the lixiviant demand rate to 40 Gt can occur if *c*_lix_ triples to 300 mM, and two of the denominator factors are reduced to ≈ ^1^/_3_^rd^ of their optimistic values (Figure 3 and Table S5).

Finally, the lixiviant demand rate can thoroughly bust the Earth’s biomass budget, exceeding net primary productivity (NPP) of 120 EJ yr^−1^ (80 Gt dry biomass) if *c*_lix_ increases to 280 mM, and all three denominator factors are reduced to ≈ ^1^/_3_^rd^ of their optimistic values (Figure 3 and Table S5).

Taken together, the results presented here suggest that CO_2_ mineralization accelerated with biologically produced lixiviants could (although this is definitely not guaranteed) place an undesirable burden on the Earth’s biosphere.

### Electromicrobial production could produce lixiviants at a cost of a few hundred dollars per tonne

Electromicrobial production technologies already have lab-scale efficiencies that can exceed the theoretical upper limit efficiencies of most forms of photosynthesis (Haas et al., 2018; Liu et al., 2016; Torella et al., 2015), and have even further room to improve (Salimijazi et al., 2020; Wise et al., 2021). This means that electromicrobial production might be able to produce lixiviants for CO_2_ mineralization from electricity and CO_2_ without needing to compete for land with agriculture and wilderness.

**Figure 4:**
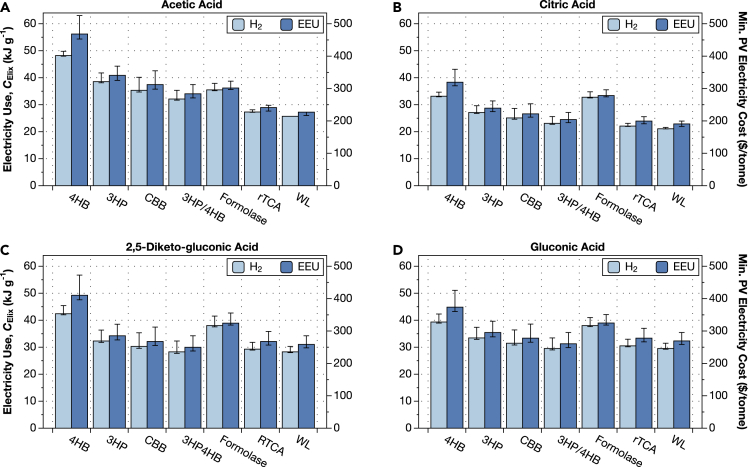
Electromicrobial production technology could reduce the electrical energy costs of lixiviant production to a few tens of kilojoules per gram (A–D) Energy and financial costs for producing four lixiviant molecules are shown in each panel: (A) acetic acid, (B) citric acid, (C) 2,5-diketo-gluconic acid (DKG), and (D) gluconic acid. The electrical energy cost of producing a gram of each lixiviant is shown on left-hand side *y* axis for each sub-plot. The dollar cost of producing a tonne of the lixiviant using electricity supplied by solar photovoltaics at a cost of 3¢ per kWh (the US Department of Energy’s cost target for solar electricity for 2030 (SunShot 2030, 2016)). This plot can be reproduced using the efficiency.py code in the ElectroCO2 repository (Barstow, 2021). The upper error bars correspond to Δ*U*_membrane_ = 240 mV, lower bars to 80 mV, and the center to 140 mV.

We used our theory of electromicrobial production (**Theory**; Salimijazi et al., 2020; Wise et al., 2021) to calculate the minimum electricity needs, and hence minimum solar electricity costs needed to produce a tonne of four different lixiviant compounds: acetic acid, citric acid, 2,5-diketogluconic acid, and gluconic acid (Figure 4).

The most expensive lixiviant to synthesize is acetic acid produced with the 4HB CO_2_-fixation pathway and with electrons supplied with extracellular electron uptake (EEU) at a cost of $56.2_{-1.9}^{+6.8}\;{\text{kJ g}}^{-1}$. Assuming that the US Department of Energy’s solar PV electricity price projection for 2030 of 3 ¢ per kilowatt-hour can be achieved, this translates to a cost of $468 per tonne of acetic acid (right-hand side axes in Figure 4).

As in our earlier analyses (Salimijazi et al., 2020; Wise et al., 2021) modifying the CO_2_ fixation method from the least efficient (the 4HB pathway) to the most efficient (the Wood-Ljungdahl pathway) can reduce the energy costs of electromicrobial production by almost a factor of 2 (Salimijazi et al., 2020; Wise et al., 2021). Likewise, switching the electron delivery mechanism to H_2_-oxidation further reduces the energy costs of production. The lowest cost method for producing acetic acid is with the Wood-Ljungdahl CO_2_-fixation pathway and with electrons supplied by H_2_-oxidation, which results in a cost of $25.7_{-0}^{+0}\;{\text{kJ g}}^{-1}$, or $214 per tonne. The lowest cost lixiviant is citric acid, with a minimum cost of $21.1_{-0.5}^{+0.1}\;{\text{kJ g}}^{-1}$ ($175 per tonne) when produced with the Wood-Ljungdahl pathway and with electron delivery by H_2_-oxidation.

Electromicrobial lixiviant production is more expensive than biomass production, even with projected 2030 solar PV prices, but might still achieve cost parity. The farm gate cost of cellulosic biomass ranges from $39.7/dry tonne for loblolly pine wood chip to $72.3/dry tonne for switchgrass (Lu et al., 2015), between 3 and 10 times cheaper than electromicrobially produced lixiviants. However, these costs do not include the cost of conversion of cellulosic biomass to a lixiviant. It is estimated that the production cost of cellulosic ethanol is $2.65 per US gallon ($890 per tonne), and it is reasonable to assume that lixiviant production would incur similar costs. Electromicrobial production of lixiviants could still achieve cost parity with biomass-derived lixiviants by directly producing the lixiviant and avoiding conversion costs.

### Electromicrobially produced lixiviants might enable cost-competitive CO_2_ mineralization

**Figure 5:**
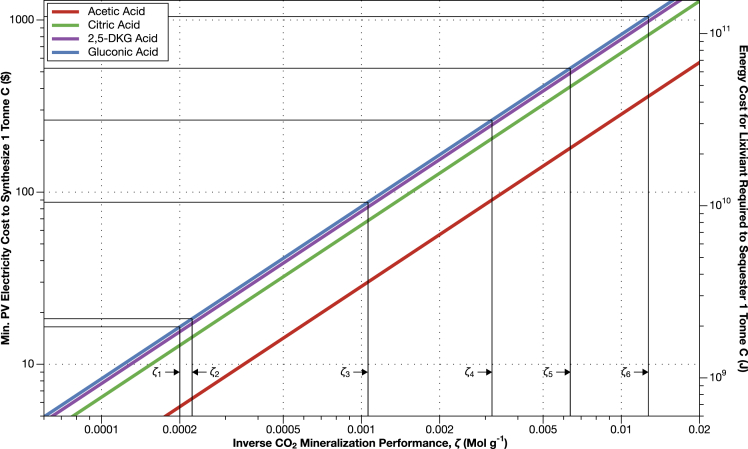
Electromicrobial production technology could enable the production of enough lixiviants to sequester 1 tonne of CO_2_ for less than $100 We combined our lixiviant mass requirements from Figure 3, with our estimates for the energy and financial cost of producing a tonne of each lixiviant compound with H_2_-mediated EMP using CO_2_-fixation with the Calvin cycle (basically the Bionic Leaf configuration (Liu et al., 2016; Torella et al., 2015)) from Figure 4. For illustrative purposes, we have marked the values of the inverse CO_2_ mineralization performance (*ζ*_1_ to *ζ*_*6*_) highlighted in Figure 3, and the corresponding cost to sequester a tonne of CO_2_ as an intersecting horizontal line. However, it is important to note that in this case, no cellulosic biomass is produced. This plot can be reproduced using the clixiviant.py code in the electroCO2 repository (Barstow, 2021).

The costs of CO_2_ mineralization with electromicrobially produced lixiviants are high, but could still enable cost-effective CO_2_ mineralization. We have plotted the amount of energy needed to synthesize enough acetic, gluconic, citric, and 2,5-diketo-gluconic acid to sequester 1 tonne of CO_2_ as a function of the inverse CO_2_ mineralization performance, *ζ*, in Figure 5. Although acetic acid is the most expensive lixiviant to produce on a per tonne basis, for a given value of *ζ*, it produces the lowest cost CO_2_ mineralization.

For the most optimistic value of *ζ* (2 × 10^−4^ Mol g^−1^), the cost of electricity (at projected 2030 PV prices) needed to make enough gluconic acid to sequester 1 tonne of CO_2_ is $17 (and only $6 for acetic acid) (Figure 5). Even when *ζ* rises to 1 × 10^−3^ Mol g^−1^ (corresponding to biomass drain from the biosphere that would prompt significant changes in global agriculture) the cost of sequestering a tonne of CO_2_ only rises to $87 when using gluconic acid, and $30 when using acetic acid (Figure 5).

These costs of CO_2_ mineralization are low enough that room could be left in the budget (the Carbon Negative Shot’s target of $100 per tonne of CO_2_) for the pre-concentration of CO_2_ with Direct Air Capture (DAC). Lackner et al. note that while DAC today is unfeasibly expensive ($500 to $600 per tonne of CO_2_), relatively modest research and development expenditure could put the technology on the cost reduction trajectory that would bring the cost to ≈ $50 per tonne (Lackner and Azarabadi, 2021). Thus, in many of the scenarios we discuss, the total cost of DAC and electromicrobially accelerated CO_2_ mineralization could be kept below $100 per tonne.

## Discussion

CO_2_ sequestration at the scale discussed in this article (20 GtCO_2_ yr^−1^) is not likely to be needed for approximately 50 years from the time of writing (around 2070). This means that there is time to identify technologies that could meet this need and refine them to do it. Weathering of ultramafic rocks and subsequent mineralization of CO_2_ almost certainly has the capacity to deal with the excess CO_2_ in the atmosphere, but accelerating this process remains a challenge.

Accelerating the weathering of ultramafic materials to the rate necessary to keep climate change within acceptable limits with organic lixiviants made from cellulosic biomass has the potential to monopolize the world’s biomass supply. Even under the most optimistic estimate of CO_2_ mineralization performance, sequestration of 20 GtCO_2_ per year could use 90% of the biomass production of the entire United States (Figure 3). If the CO_2_ mineralization performance were to slip even slightly, accelerated CO_2_ mineralization could force undesirable changes in the world agricultural system and society (Figure 3).

Electromicrobial production of organic lixiviants could enable accelerated CO_2_ mineralization without competing for agricultural land. Although EMP technologies only exist in the lab today, their high lab scale and even higher predicted maximum solar to product conversion efficiencies mean that they could be an effective tool in CO_2_ management. In this article, we demonstrate that organic lixiviants can be produced by EMP at the cost of ≈ $200 to $400 per tonne assuming solar electricity is supplied at a cost of 3¢ per kWh (a target for 2030 solar electricity costs set by the US Department of Energy (SunShot 2030, 2016)) (Figure 4).

Electromicrobially produced lixiviants could enable large-scale CO_2_ mineralization at low costs. We show that even with modest CO_2_ mineralization performance, the cost of making the lixiviants needed to sequester a tonne of CO_2_ could be kept below $100 per tonne, even with 2030 solar electricity costs (Figure 5). It is highly likely that many more halvings of solar electricity costs will occur between 2030 and 2070, further reducing the cost of CO_2_ mineralization. We believe that the analysis presented here shows that testing our predictions of the efficiency of lixiviant production from renewable electricity and CO_2_ at lab scale is definitely worth pursuing.

Can these costs be achieved in reality? Several scientific and engineering questions need to be answered to assess this. First, does a lixiviant produced by EMP need to be purified, or is a whole cell culture required to achieve high-efficiency mineral dissolution? If the purification of the lixiviant is required, what cost does this impose on the process? Biolixiviants appear to contain many more compounds than just acids that dramatically increase their potency (Reed et al., 2016). Can we reprogram the cell to release these prior to even seeing a rock, so that the lixiviant can be used in a cell-free form?

On the other hand, if a whole-cell culture has to be used for mineral dissolution, how can the escape of genetically modified organisms into the environment, especially given the enormous scale of CO_2_ sequestration, be prevented? Even if this process were to occur on the surface of the Earth in an environment similar to a mine, this presents enormous challenges for biocontainment. We anticipate that if engineered organisms are used for CO_2_ sequestration this will require an extensive overhaul of the government regulation of synthetic biology on one hand, and extensive use of advanced bio-containment technologies such as engineered auxotrophies (Rovner et al., 2015) on the other hand. We hope that the potential for this lixiviant accelerated CO_2_ mineralization process shown in this article inspires others to pursue these questions.

What’s the best way to achieve the potential of EMP for CO_2_ mineralization? Until recently, the difficulty of adding CO_2_ fixation to a non-CO_2_-fixing organism; uncertainty about the efficiency, and even nature of electron uptake; and the difficulty of engineering non-model organisms such as the mineral-dissolving microbe *G. oxydans* have made a project like this look unfeasible. However, recent developments make this look increasingly possible. Gleizer et al., 2019 transformed the lab workhouse microbe *Escherichia coli* to fix CO_2_, while Yishai et al., 2017 and Tashiro et al., 2018 have both demonstrated formate assimilation by engineered *E. coli*, and Kim et al. have demonstrated the growth of engineered *E. coli* on formate (Kim et al., 2020). Rowe et al., 2018 discovered that *S. oneidensis* can use imported electrons to reduce NADH, and characterized the genes behind this pathway (Rowe et al., 2021). Schmitz et al. recently built a whole-genome map of acid production by *G. oxydans* (Schmitz et al., 2021), the first step in whole genome engineering. Added together these breakthroughs make something that appeared almost impossible a few years ago, appear tantalizingly possible.

### Limitations of the study

This article proposes a high-level overview of the costs to the biosphere (i.e., how much biomass will need to be diverted from agriculture and ecological services) of using biological lixiviants to accelerate carbon mineralization. However, we find that there is significant uncertainty surrounding the amount of lixiviant needed to sequester a given amount of CO_2_. But, this study estimates that the production of biological lixiviants needed to sequester 20 gigatonnes of CO_2_ per year (the IPCC’s estimate for the amount of CO_2_ needed to be withdrawn to maintain global temperatures by the end of the century) could easily monopolize a significant fraction of global agricultural output except in the most optimistic scenarios. The study highlights the potential benefits (i.e., significantly reduced competition for land) of producing biolixiviants with genetically engineered carbon-fixing electroactive microbes (electromicrobial production) that can operate at efficiencies much greater than natural photosynthesis. However, the feasibility of achieving anywhere near the upper limit efficiencies of electromicrobial production used in this article remains to be determined, as do the costs of deploying this technology. The purpose of this article is to build interest and support for further research into biolixiviant production with engineered microbes.

## STAR★Methods

### Key resources table

**Table:** 

REAGENT or RESOURCE	SOURCE	IDENTIFIER
**Software and algorithms**		

Python 3.9.6	Python Software Foundation	https://www.python.org
iPython 7.2.6.0	The iPython Development Team	https://www.ipython.org
Model code	This paper	https://doi.org/10.5281/zenodo.5805345 https://github.com/barstowlab/electroco2

### Resource availability

#### Lead contact

Further information and requests for resources and reagents should be directed to and will be fulfilled by the lead contact, Buz Barstow (bmb35@cornell.edu).

#### Materials availability

This study did not generate any unique reagents.

### Method details

All computer models were performed with iPython version 7.26.0 with Python 3.9.6 (for details, see key resources table). Graphs were produced with DataGraph, and graphics were produced with Adobe Illustrator.

#### Calculation of lixiviant pH

In order calculate the pH of a lixiviant at a given concentration for use in Figure 3, we adapted this calculation from (Helmenstine, 2019 ) that asks what is the pH of a weak organic acid, with a given pKa, at a particular analytical concentration?

The association constant, K_a_,

(Equation 17) $${\text{K}}_{\text{a}}=\left[\text{H}+\right]\left[\text{B}-\right]/\left[\text{HB}\right]$$

where [H^+^] is concentration of H^+^ ions, [B^-^] is concentration of conjugate base ions, and [HB] is the concentration of undissociated acid molecules. We assume that the acid releases one H^+^ ion for every B^-^ ion, so

(Equation 18) $$\left[{\text{H}}^{+}\right]=\left[{\text{B}}^{-}\right].$$

To simplify the algebra, we denote [H^+^] as *x.* Thus,

[HB]=C−x,

where *C* is the analytical concentration of the acid. Thus, using Equation 17,

(Equation 19) $$K_{a}=\frac{x\times x}{C-x},$$

(Equation 20) $$x^{2}=K_{a}\left(C-x\right),$$

(Equation 21) $$x^{2}+K_{a}x-CK_{a}=0.$$

The proton concentration, *x,* can be found using the positive root of the quadratic equation,

(Equation 22) $$x=-\frac{b}{2a}\pm \frac{1}{2a}\sqrt{b^{2}-4ac},$$

(Equation 23) $$x=-\frac{K_{a}}{2}\pm \frac{1}{2}\sqrt{K_{a}^{2}+4CK_{a}}.$$

Thus,

(Equation 24) $$\text{pH}=-{\text{log}}_{10}\left(-\frac{K_{a}}{2}+\frac{1}{2}\sqrt{K_{a}^{2}+4CK_{a}}\right).$$

For acetic acid (pK_a_ = 4.75), citric acid (pK_a_ = 3.13), and gluconic acid (pK_a_ = 3.72) at 100 mM,

(Equation 25) $${\text{pH}}_{\text{acetic}}=2.9,$$

(Equation 26) $${\text{pH}}_{\text{gluconic}}=2.4,$$

(Equation 27) $${\text{pH}}_{\text{citric}}=2.1.$$

## Data Availability

All original code has been deposited at Github and archived at Zenodo (Barstow, 2021) and is publicly available as of the date of publication. DOIs are listed in the key resources table.

## Supplemental information

Document S1. Tables S1–S5

Data S1. Enzymatic reactions for the synthesis of lixiviant compounds from CO2 or formic acid, related to Figure 4
