# Supplementary material for: Practical and thermodynamic constraints on electromicrobially accelerated CO2 mineralization
Source: iScience. 2022 Jul 16;25(8):104769. doi: 10.1016/j.isci.2022.104769 (PMC9385556; doi:10.1016/j.isci.2022.104769)
Supplement: Document S1. Tables S1–S5 [file mmc1.pdf]

iScience, Volume *25*

Supplemental information

Practical and thermodynamic constraints on electromicrobially accelerated CO_2 mineralization

Sabrina Marecos, Rae Brigham, Anastacia Dressel, Larissa Gaul, Linda Li, Krishnathreya Satish, Indira Tjokorda, Jian Zheng, Alexa M. Schmitz, and Buz Barstow

Supplementary Information for:

Practical and Thermodynamic Constraints on Electromicrobially-Accelerated CO_2 Mineralization

Sabrina Marecos[1], Rae Brigham[1], Anastacia Dressel[1]*, Larissa Gaul[1]*, Linda Li[1]*, Krishnathreya Satish[1]*, Indira Tjokorda[1]*, Jian Zheng[1]*, and Buz Barstow[1†]

[1]Department of Biological and Environmental Engineering, Cornell University, Ithaca, NY 14853, USA

[*]These authors contributed equally to this article.

†Corresponding author:

Buz Barstow, 228 Riley-Robb Hall, Cornell University, Ithaca, NY 14853; bmb35@cornell.edu

**Lixiviant Compound**	**Molecular Weight (Da)**	**Molecular Formula**	**Carbons per Molecule**
Acetic Acid	60.052	CH_3COOH	2
Citric Acid	192.124	$C_6H_8O_7$	6
2,5-Diketo-Gluconic Acid	191.12	$C_6H_8O_7$	6
Gluconic Acid	196.16	$C_6H_{12}O_7$	6
Glucose	180.16	$C_6H_{12}O_6$	6

Table S1. Molecular weights and energy densities for lixiviant molecules, related to **Figures 4** and **5**.

**Reaction**	**Reference**
1. Acetic Acid	
Acetyl-CoA + ADP + phosphate → Acetate + ATP + CoA	KEGG R0229
2. Citric Acid	
Pyruvate + CO_2 → Oxaloacetate	KEGG RC00040
Acetyl-CoA + H_2O + Oxaloacetate → Citrate + CoA	KEGG RC00351
3. 2,5-Diketo-Gluconic Acid	
ATP + Pyruvate + HCO_3^- → Orthophosphate + Oxaloacetate	KEGG R00344
ATP + Oxaloacetate → Phosphoenolpyruvate + CO_2	KEGG R00341
Phosphoenolpyruvate + H_2O → 2-Phospho-D-glycerate	KEGG R00658
2-Phospho-D-glycerate → 3-Phospho-D-glycerate	KEGG R01518
ATP + 3-Phospho-D-glycerate → 3-Phospho-D-glyceroyl phosphate	KEGG R01512
3-Phospho-D-glyceroyl phosphate + NADH + H^+ → D-Glyceraldehyde 3-phosphate + Orthophosphate	KEGG R01061
D-Glyceraldehyde 3-phosphate → Glycerone phosphate	KEGG R01015
Glycerone phosphate + D-Glyceraldehyde 3-phosphate → D-Fructose 1,6-bisphosphate	KEGG R01068
D-Fructose 1,6-bisphosphate + H_2O → D-Fructose 6-phosphate + Orthophosphate	KEGG R00762
D-Fructose 6-phosphate → D-Glucose 6-phosphate	KEGG R00771
D-Glucose 6-phosphate + H_2O → D-Glucose + Orthophosphate	KEGG R00303
beta-D-Glucose → D-Glucono-1,5-lactone + NADH+ H^+	KEGG R01521
D-Glucono-1,5-lactone + H_2O → D-Gluconate	KEGG R01519
D-Gluconate → 2-Keto-D-gluconic acid + H^+	KEGG R01739
2-Keto-D-gluconic acid → 2,5-Diketo-D-gluconic acid+ H^+ + NADPH	KEGG R05823
4. Gluconic Acid	
ATP + Pyruvate + HCO_3^- → ADP + Phosphate + Oxaloacetate	KEGG R00344
ATP + Oxaloacetate → ADP + Phosphoenolpyruvate + CO_2	KEGG R00341
Phosphoenolpyruvate + H_2O → 2-phospho-D-glycerate	KEGG R00658
2-phospho-D-glycerate → 3-phospho-D-glycerate	KEGG R01518
ATP + 3-phospho-D-glycerate → ADP + 3-phospho-D-glyceroyl phosphate	KEGG R01512
3-phospho-D-glyceroyl phosphate + NADH + H^+ → D-glyceraldehyde 3-phosphate + Phosphate + NAD^+	KEGG R01061
D-glyceraldehyde 3-phosphate → Glycerone phosphate	KEGG R01015
Glycerone phosphate + D-glyceraldehyde 3-phosphate → D-fructose 1,6-bisphosphate	KEGG R01068
D-fructose 1,6-bisphosphate + H_2O → D-fructose 6-phosphate + Phosphate	KEGG R00762
D-fructose 6-phosphate → D-glucose 6-phosphate	KEGG R00771
D-glucose 6-phosphate + H_2O → D-glucose + Phosphate	KEGG R00303
D-glucose + $NAD(P)^+$ → D-glucono-1,5-lactone + NAD(P)H + H^+	KEGG R01520
D-glucono-1,5-lactone + H_2O → D-gluconate	KEGG R01519

Table S2. Reactions for synthesis of lixiviant molecules, related to **Figure 4**. Reactions for lixiviant synthesis from acetyl-CoA, NAD(P)H, Ferredoxin and ATP were assembled from data from the KEGG database (Kanehisa and Goto, 2000; Kanehisa *et al.*, 2019; Kanehisa *et al.*, 2021).

Reaction	Reference
1. Calvin Cycle (CBB)	
2 CO_2 + 7 ATP + 4 NADH → 1 Acetyl-CoA	Salimijazi *et al*. (Salimijazi *et al*., 2020).
3 CO_2 + 7 ATP + 5 NADH → 1 Pyruvate	Salimijazi *et al.* (Salimijazi *et al*., 2020).
2. Wood-Ljungdahl Pathway (WL)	
4 CO_2 + 2 ATP + 8 NADH → 2 Acetyl-CoA	Berg (Berg *et al.*, 2011)
2 Fd_{red} + Acetyl-CoA + CO_2 → Pyruvate	KEGG R01196.
3. Reductive TCA Cycle (RTCA)	
4 CO2 + 4 ATP + 8 NADH → 2 Acetyl-CoA	(Alissandratos *et al.*, 2015; Claassens *et al*., 2016).
2 Fd_{red} + Acetyl-CoA + CO_2 → Pyruvate	KEGG R01196.
4. 3-hydroxypropionate/4-hydroxybutyrate Cycle (3HP4HB)	
6 HCO_3^- + 10 ATP + 10 NADH → 2 pyruvate	(Berg *et al*., 2007a; Claassens *et al*., 2016).
2 Pyruvate → 2 Acetyl-CoA + 2 NADH + 2 CO_2	(Berg *et. al*, 2002; Schomburg *et al.* 2017).
5. 3-hydroxypropionate Cycle (3HP)	
6 HCO_3^- + 10 ATP + 12 NADH → 2 Pyruvate	(Zarzycki *et al*., 2009; Herter *et al*., 2002; Berg *et. al*, 2002).
2 Pyruvate → 2 Acetyl-CoA + 2 NADH + 2 CO_2	(Zarzycki *et al*., 2009; Herter *et al*., 2002; Berg *et. al*, 2002).
6. 4-hydroxybutyrate Cycle (4HB)	
1 CO_2 + 1 HCO_3^- + 3 ATP + 1 NADH + 6 Fd_{red} → 1 Acetyl-CoA	(Huber *et al*., 2008).
2 Pyruvate → 2 Acetyl-CoA + 2 NADH + 2 CO_2	(Berg *et. al*, 2002; Schomburg *et al.* 2017).
7. Formolase Pathway (FORM)	
6 HCO_2^- + 10 ATP + 4 NADH → 2 3-PG	(Siegel *et al*., 2015; Bar-Even *et al*., 2016).
2 3-PG → 2 Pyruvate + 2 ATP	(Berg *et. al*, 2002).
2 Pyruvate → 2 Acetyl-CoA + 2 NADH + 2 CO_2	(Berg *et. al*, 2002; Schomburg *et al.* 2017).

Table S3. CO_2-fixation and C_1-assimilation reactions. CO_2-fixation and C_1-assimilation reactions, related to **Figure 4**. Reactions in this article were first assembled in Salimijazi *et al*., 2020 and are restated here for convenience. Overall reactions for production of metabolic intermediates by 6 naturally-occurring CO_2-fixation cycles and the synthetic Formolase formate assimilation pathway, and the FeMoCo nitrogenase N_2-fixation reaction. Reactions can be referenced KEGG database (Kanehisa *et al*., 2000; Kanehisa *et al.*, 2019; Kanehisa *et al*., 2021). Fd_{red}: Reduced Ferredoxin; 3-PG: 3-Phosphoglycerate.

Scenario	ATP	NAD(P)H	Fd_{red}	CO_2	HCO_3^-	HCO_2^-	Total C	Target Molecule	Target Formula	Target
Acetic_3HP	4	5	0	-1	3	0	2	Acetate	CH_3COOH	1.0
Acetic_3HP4HB	4	4	0	-1	3	0	2	Acetate	CH_3COOH	1.0
Acetic_4HB	2	1	6	1	1	0	2	Acetate	CH_3COOH	1.0
Acetic_CBB	6	4	0	2	0	0	2	Acetate	CH_3COOH	1.0
Acetic_FORM	3	1	0	-1	0	3	2	Acetate	CH_3COOH	1.0
Acetic_RTCA	1	4	-0	2	0	0	2	Acetate	CH_3COOH	1.0
Acetic_WL	-0	4	-0	2	0	0	2	Acetate	CH_3COOH	1.0
Citric_3HP	10	11	0	0	6	0	6	Citrate	$C_6H_8O_7$	1.0
Citric_3HP4HB	10	9	0	0	6	0	6	Citrate	$C_6H_8O_7$	1.0
Citric_4HB	6	3	12	4	2	0	6	Citrate	$C_6H_8O_7$	1.0
Citric_CBB	14	9	0	6	0	0	6	Citrate	$C_6H_8O_7$	1.0
Citric_FORM	8	3	0	0	0	6	6	Citrate	$C_6H_8O_7$	1.0
Citric_RTCA	4	8	2	6	0	0	6	Citrate	$C_6H_8O_7$	1.0
Citric_WL	2	8	2	6	0	0	6	Citrate	$C_6H_8O_7$	1.0
DKG_3HP	16	12	0	-2	8	0	6	2 5-DKG	$C_6H_8O_7$	1.0
DKG_3HP4HB	16	10	0	-2	8	0	6	2 5-DKG	$C_6H_8O_7$	1.0
DKG_4HB	12	2	16	2	4	0	6	2 5-DKG	$C_6H_8O_7$	1.0
DKG_CBB	20	10	0	4	2	0	6	2 5-DKG	$C_6H_8O_7$	1.0
DKG_FORM	14	4	0	-2	2	6	6	2 5-DKG	$C_6H_8O_7$	1.0
DKG_RTCA	10	8	4	4	2	0	6	2 5-DKG	$C_6H_8O_7$	1.0
DKG_WL	8	8	4	4	2	0	6	2 5-DKG	$C_6H_8O_7$	1.0
Gluconate_3HP	16	13	0	-2	8	0	6	D-Gluconate	$C_6H_{12}O_7$	1.0
Gluconate_3HP4HB	16	11	0	-2	8	0	6	D-Gluconate	$C_6H_{12}O_7$	1.0
Gluconate_4HB	12	5	12	2	4	0	6	D-Gluconate	$C_6H_{12}O_7$	1.0
Gluconate_CBB	20	11	0	4	2	0	6	D-Gluconate	$C_6H_{12}O_7$	1.0
Gluconate_FORM	12	5	0	0	0	6	6	D-Gluconate	$C_6H_{12}O_7$	1.0
Gluconate_RTCA	10	9	4	4	2	0	6	D-Gluconate	$C_6H_{12}O_7$	1.0
Gluconate_WL	8	9	4	4	2	0	6	D-Gluconate	$C_6H_{12}O_7$	1.0

Table S4. Net molecular input requirements for lixiviant synthesis by 6 naturally-occurring CO_2-fixation cycles and the synthetic Formolase formate assimilation pathway, related to **Figure 4**. Fd_{red}: Reduced Ferredoxin. 3HP: 3-hydroxypropionate cycle (Zarzycki *et. al*, 2009); 3HP4HB: 3-hydroxypropionate/4-hydroxybutyrate pathway (Berg *et al.,* 2007; Claassens *et al.,* 2016); 4HB: Dicarboxylate/4-hydroxybutyrate cycle (Huber *et al.,* 2008); Calvin-Benson-Bassham cycle (Berg *et al.,* 2002); FORM: Formolase formate assimilation pathway (Siegel *et al.,* 2015a); RTCA: Reductive Tricarboxylic Acid cycle (Alissandratos *et al.,* 2015; Claassens *et al.,* 2016); WL: Wood-Ljungdahl (WL) Pathway (Berg *et al.,* 2002). Results can be reproduced running the BALANCE.PY code in the ELECTROCO2 repository (**Key Resources Table**).

#	Inverse CO_2 Mineralization Economy, ζ (Mol g^{-1})	Lixiviant Concentration, c_{lix} (Moles per m^3 or mM)	Extraction Efficiency, η_{ex}	Precipitation Efficiency, η_{precip}	Pulp Density, ρ_{pulp} (grams per m^3)	Note
ζ_1	2.00×10^{-4}	100.0	1.00	1.00	5.00×10^5	Our most optimistic estimate of inverse CO_2 mineralization economy, ζ.
ζ_2	2.23×10^{-4}	100.0	1.00	0.90	5.00×10^5	Value of ζ corresponding to use of entire US biomass production.
		111.5	1.00	1.00	5.00×10^5	
		100.0	0.90	1.00	5.00×10^5	
		102.8	0.97	0.97	4.87×10^5	
ζ_3	1.06×10^{-3}	100.0	1.00	0.19	5.00×10^5	Value of ζ corresponding to first global agricultural transition identified by Slade *et al*., 2014.
		100.0	0.19	1.00	5.00×10^5	
		530.0	1.00	1.00	5.00×10^5	
		100.0	1.00	1.00	9.40×10^4	
		100.0	0.44	0.44	5.00×10^5	
		229.9	1.00	1.00	2.18×10^5	
		151.7	0.66	0.66	3.30×10^5	
ζ_4	3.18×10^{-3}	100.0	0.25	0.25	5.00×10^5	Value of ζ corresponding to second global agricultural transition identified by Slade *et al*., 2014.
		398.9	1.00	1.00	1.25×10^5	
		199.8	0.50	0.50	2.50×10^5	
ζ_5	6.36×10^{-3}	237.5	0.42	0.42	2.10×10^5	Value of ζ corresponding to third global agricultural transition identified by Slade *et al*., 2014.
		316.8	0.32	0.32	5.00×10^5	
		316.8	1.00	0.32	1.58×10^5	
ζ_6	1.27×10^{-2}	282.5	0.35	0.35	1.77×10^5	Value of ζ corresponding to use of entire global net primary production identified by Slade *et al*., 2014.

Table S5. Possible combinations of factors that produce significant values of inverse CO_2 mineralization performance, ζ, related to **Figure 2**. We calculated possible combinations of values of c_{lix}, η_{ex}, η_{precip}, and ρ_{pulp} that produce each of the values of ζ highlighted in **Figure 2**.
